# The effects of *Arabidopsis* genome duplication on the chromatin organization and transcriptional regulation

**DOI:** 10.1093/nar/gkz511

**Published:** 2019-06-11

**Authors:** Hui Zhang, Ruiqin Zheng, Yunlong Wang, Yu Zhang, Ping Hong, Yaping Fang, Guoliang Li, Yuda Fang

**Affiliations:** 1 National Key Laboratory of Plant Molecular Genetics, CAS Center for Excellence in Molecular Plant Sciences, Institute of Plant Physiology and Ecology, Chinese Academy of Sciences; University of Chinese Academy of Sciences, Shanghai 200032, China; 2 National Key Laboratory of Crop Genetic Improvement, Hubei Key Laboratory of Agricultural Bioinformatics, Hubei Engineering Technology Research Center of Agricultural Big Data, College of Informatics, Huazhong Agricultural University, Wuhan 430070, China

## Abstract

Autopolyploidy is widespread in higher plants and important for agricultural yield and quality. However, the effects of genome duplication on the chromatin organization and transcriptional regulation are largely unknown in plants. Using High-throughput Chromosome Conformation Capture (Hi-C), we showed that autotetraploid Arabidopsis presented more inter-chromosomal interactions and fewer short-range chromatin interactions compared with its diploid progenitor. In addition, genome duplication contributed to the switching of some loose and compact structure domains with altered H3K4me3 and H3K27me3 histone modification status. 539 genes were identified with altered transcriptions and chromatin interactions in autotetraploid Arabidopsis. Especially, we found that genome duplication changed chromatin looping and H3K27me3 histone modification in *Flowering Locus C*. We propose that genome doubling modulates the transcription genome-wide by changed chromatin interactions and at the specific locus by altered chromatin loops and histone modifications.

## INTRODUCTION

Polyploidy plays a significant role in plant evolution and formation of the important agriculture traits such as high yield and stress resistances in crops (1–3). Polyploidy crops own higher plasticity and environmental adaptability (4) with specific gene expression patterns for the organ development and resistances to abiotic and biotic stresses (1,5).

Recent studies have illustrated that nuclear architecture is important for gene expression and recombination (6–8), and the distribution and condensation of chromatin fiber could be influenced by various environmental and physical factors (9,10). Most eukaryotic genomes are organized at hierarchical levels (11–13). First, each chromosome has a preferred but not fixed position named chromosome territory (CT) (14), which consist of several megabase-scale genomic compartments (15,16). These compartments are categorized into A-type compartment rich for euchromatic and highly transcribed regions and B-type compartment rich for heterochromatic and gene-poor regions (17). Further, within the genomic compartments, topologically associating domains (TADs) were identified as clusters of chromatin interactions with their boundaries associated with architecture proteins like CTCF and cohesin, and activating histone markers (18–20). Moreover, the smaller scale structures like chromatin loops at 10 kb to 1 Mb can appear within the topological domains (21,22). Chromatin looping could bring cis-regulatory elements and promoters together to affect gene transcription (23).

Studies on the three-dimensional (3D) genome organization have proposed that Arabidopsis exhibited no large local interactive domains (24). The chromosome territories of Arabidopsis are organized as chromosome-arm territories which set centromeres as strong insulators that block the interactions across chromosome arms (25). Arabidopsis does not form obvious TADs, which is different from animals and other large genome plants (26–28). In addition, the Arabidopsis chromosome arms are partitioned into loose structural domain (LSD) and compacted structural domain (CSD) which are equivalent to A- and B-type compartments in animals (29). The studies on the relationships between chromatin packing and epigenetic modifications in mutants defective in the specific epigenetic pathway suggested that changes in the epigenome could correlate with chromatin interaction patterns (30). The chromatin packing in diploid and allotetraploid cottons with large genomes was shown to be modulated by the density of chromatin and chromosome number (26). Genome doubling in rice resulted in changes of DNA methylome of transposable elements and transcriptome (31). However, little is known about the relationship between genome organization and gene regulation in autopolyploid plants.

To address the spatial arrangement of the doubled genome and the relationship between chromatin structure and transcriptional regulation, we performed High-throughput Chromosome Conformation Capture (Hi-C) analysis of the autotetraploid (genome-duplicated) and diploid Arabidopsis. We showed that the genome duplication resulted in increased inter-chromosome interactions and decreased intra-chromosome arm interactions compared with its diploid progenitor. In conjunction with RNA-seq and ChIP-seq, we found that the transcription of a group of genes is specifically affected by the chromatin interactions with epigenome dynamics. These results shed light on the 3D genomic organization and transcriptional regulation of the autotetraploid plants.

## MATERIALS AND METHODS

### Plant materials

*Arabidopsis thaliana* accession Columbia (Col-0) was used as wild type. Autotetraploid Arabidopsis (4 × Col-0) generated by colchicine using Col-0 as the progenitor (32) was a kind gift from Prof. Daiyin Chao (4), SIPPE. Here the autotetraploid Arabidopsis represents the genome-duplicated line. Plants were grown at 23°C in long day condition (16 h light and 8 h dark cycles) on Murashige & Skoog (MS) medium supplemented with 1% sucrose and 0.8% agar. After stratification at 4°C for 3 days, the plants were transferred to the long day condition for 10 days, and then the aerial parts of these seedlings were harvested for Hi-C, ChIP-seq and RNA-seq library preparation.

### Flow cytometry

Ten-day-old Col-0 or 4 × Col-0 Arabidopsis seedlings were collected into a clean and pre-cooled plate, and then chopped with a new razor blade to release the nuclei in the sterile lysis buffer (45 mM MgCl_2_·6H_2_O, 30 mM sodium citrate (trisodium), 20 mM MOPS, 1% Triton-100, pH 7.0) for 3–5 min until the buffer turns green. Transfer the mixture into a 40 μm strainer. The filtrates were added DAPI solution with final concentration of 1 ng/ml, then incubate for 30 min in the dark on ice. The DAPI-treated filtrates were then loaded to the flow cytometer (Beckman Coulter Moflo-XDP, USA) to measure the ploidy levels.

### Flowering time and rosette leaf number measurement

After stratification, the seeds of Col-0 and 4 × Col-0 were sown on the MS plates, and grown for 10 days. The seedlings were then transferred to the soil under long day condition until flowering. The days of flowering and rosette numbers were scored.

### Hi-C library preparation

Hi-C experiments were performed essentially as described (29) with some modifications. Briefly, 2.5 g aerial parts of the seedlings were fixed and grounded into powder in liquid nitrogen. The extracted nuclei were digested with 600 U *Hind*III restriction enzyme by incubating overnight at 37°C, then the digested chromatin was blunt-ended with 1μl 10 mM dATP, dTTP, dGTP and 25 μl 0.4 mM biotin-14-dCTP and 100 U Klenow fragment for 45 min at 37°C. Next, the ligation reaction was performed in 10 time volume of ligation production buffer under constant shaking with 745 μl 10× ligation buffer and 10% Triton X-100, 80 μl 10 mg/ml BSA and ATP and 100 Weiss U T4 DNA ligase. The ligation reaction was performed at 16°C for 6 h. After ligation, the nuclei were reverse-crosslinked with proteinase K at 65°C overnight. Subsequently, the extracted chromatin was fragmented into a mean size of 300 bp using a sonicator (Covaris S220). Hi-C libraries were constructed with NEBNext Multiplex Oligos kit and KAPA Hyper Prep Kit. The final library was subject to sequencing on an Illumina HiSeq 2000 instrument with 2 × 150-bp reads.

### Hi-C sequencing data processing

After removing the adapter, the clean Hi-C reads of Col-0 and 4 × Col-0 were aligned to *Arabidopsis* reference genome (TAIR10). Following with HiC-Pro and Juicer software (33,34), valid pairs of wild type and autotetraploid were used to create interaction matrixes with bin size 50 kb for further analysis. The interaction matrixes were normalized with KR method from Juicer (34). The reproducibility of two biological replicates was tested with Pearson correlation coefficient from the interaction matrixes (35).

After excluding the pericentromeres as reported (29), the first principal component was used to identify chromatin structure domains (SDs) with Juicer. We set the positive Eigenvalues associated with loose structure domains (LSD) and negative Eigenvalues with compacted structural domains (CSD).

### Calculation of chromatin interaction and interaction decay exponents

To analyze the difference of interaction matrixes between Col-0 and 4 × Col-0, the normalized interaction matrix from Col-0 was divided by the normalized interaction matrix from 4 × Col-0, with all the zeros in the matrixes replaced with 1% quintiles from the non-zero elements in each matrix. Log2 transformation and median normalization were used to standardize the difference matrix.

To understand the interaction frequency changes dependent on the genome distance, interaction decay exponents (IDEs) were calculated (29) for chromosomes, pericentromeres and telomeres.

### RNA-seq and data analysis

Total RNAs were extracted with RNeasy Plant Mini kit (Qiagen), and the libraries were constructed according to a standard protocol (Illumina). Sequencing reads were aligned against the reference genome (TAIR10) using Feature Counts and STAR with default parameters (36). The differential expression analysis was run using the classical normalization method DESeq2 R package (37) with a 0.05 *p*-value, 0.05 false discovery rate, and cutoff of 1 log-fold change. Gene ontology (GO) terms were extracted from clusterprofiler R package (38). All of the data were from three biological replicates.

### ChIP-seq and data analysis

The ChIP assay was performed according to previously reported (39). Briefly, 2.5 g fresh 10-day-old seedlings were cross-linked in the cross-linking buffer (0.4 M sucrose, 10 mM Tris–HCl (pH 8.0), 1 mM PMSF, 1 mM EDTA, 1% formaldehyde) for 3 × 5 min using vacuum infiltration and the reaction was terminated in 2 M glycine. Chromatin was sheared to an average size of 300 bp with a sonicator (Bioruptor, Diagenode). Then the sonicated samples were immunoprecipitated with 5 μg anti-H3K27me3 (Millipore 07-449), anti-H3K4me3 (Millipore 07-473) and anti-H3 (Abcam ab1791) antibodies. After incubation at 4°C overnight, the antibodies were recovered with 20 μl Protein A/G magnetic beads (Millipore 16-663). After reverse cross-link, ChIP-ed DNA was extracted with phenol-chloroform method and sequencing library was constructed according to the standard Illumina protocol. The library was sequenced using an Illumina HiSeq 2000 instrument. The sequencing reads were aligned to the reference genome with BWA (40). The mapped reads were analyzed using Macs2 with default parameters for peak calling (41). Reads from anti-H3 samples were set as the control.

For ChIP-PCR, the immunoprecipitated DNA template and primers covering promoter, exon and intron of *FLC* (Supplementary Table S6) were used to quantify histone modifications by real-time PCR. Real-time PCR data of H3K27me3, H3K4me3 and H3 enrichment at *FLC* locus were normalized to 1% Input.

### Histone modification quantifications on CSD and LSD

To quantify the genome wide histone modification levels in all CSDs and LSDs, the means of H3K4me3 and H3K27me3 RPKM (reads per kilobase per million mapped reads) were calculated in CSD and LSD regions in 50 kb bins in Col-0 and 4 × Col-0. Because the SDs were identified with PCA1 (Principal Component Analysis 1), a relatively broad parameter which could not reflect the more complicated structures. To analyze the relationship between histone modification and SD transition more precisely, *K*-means clustering was used to remove histone modification noise signals. In this method, we set *K* = 10 to cluster the same kind of histone modification on a given SD. Histone modification peaks in the SD were called and the RPKM in these called peaks was used to compare the differences between these SDs. Jaccard index was used to indicate the correlation between histone modification and SD.

### Fluorescence microscopy and image processing

The binary vector of *HTR12-GFP* (42) was used to obtain transgenic plants in Col-0 and 4 × Col-0 backgrounds for centromere visualization under microscopy. 10-day-old seedling leaves were subjected to microcopy analyses. Images of nuclei were acquired with the DeltaVision Personal DV system (Applied Precision). Data collection and image processing were performed according to Fang et al. (43,44). Fluorescence foci were subjected to statistical analysis using the Spot Pattern module of Imaris software (Bitplane AG).

### Bootstrapping analysis

In the bootstrapping strategies (45), 1000 groups (*n* = 1000 times) of equal number randomly selected no-regulated genes were subjected to the same analysis to determine the percentage of those groups fallen in differentially interaction bins. The percentile of the test sample lie above the top 5 percentile of the control distribution was considered confidently.

### Promoter-Promoter and Promoter-Enhancer analysis

The 2 kb upstream of TSS was selected as the promoter. The distribution of promoters was then analyzed for each bin, and the bins that contained more than the median number of promoters was defined as promoter-enriched bins. The interaction frequency among these promoter-enriched bins was calculated as the genome-wide promoter-promoter interaction frequency.

For analyzing the interactions of promoter-enhancers, the published candidate enhancer library (46) was used to identify enhancer-enriched bins. The bins which contain the number of enhancers more than the median were defined as enhancer-enriched bins. The interaction frequency between enhancer-enriched bins and promoter-enriched bins was calculated as the genome-wide promoter-enhancer interaction frequency.

### Chromosome Conformation Capture (3C) test

Two grams of 10-day-old seedlings were cross-linked and nuclei were isolated as previously reported (29). Digestion was performed with 400 U *Bam*HI and *Bgl*II at 37°C overnight. After inactivating the enzyme with SDS, the ligation reaction was performed by incubation at 16°C for at least 6 h with 100 U T4 DNA ligase. Reverse cross-link was performed at 65°C by treatment with Proteinase K overnight. Relative interaction frequency was assessed by real-time PCR (47).

## RESULTS

### Phenotypic and transcriptomic changes caused by Arabidopsis genome doubling

**Figure 1.**
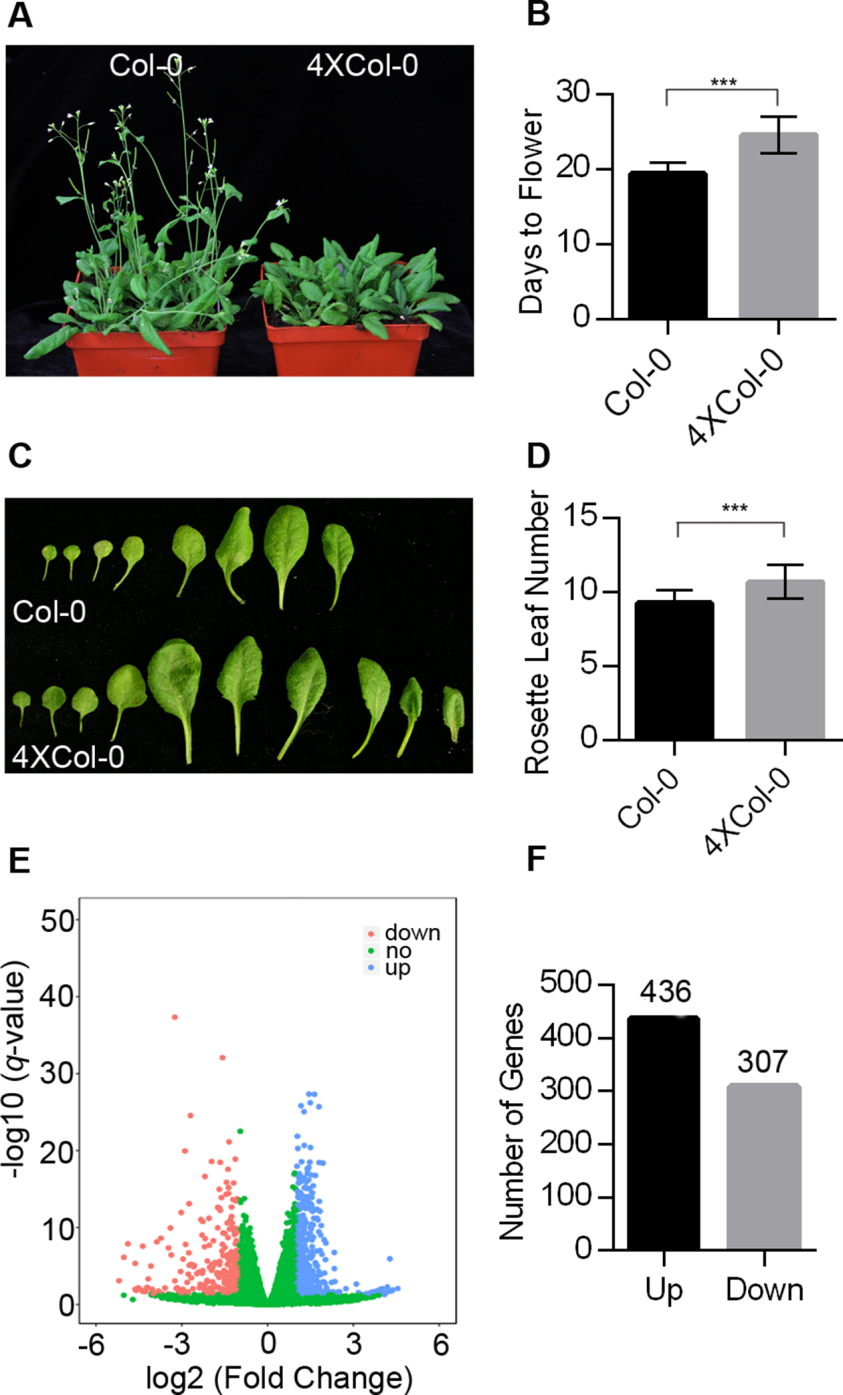
Comparison of morphology and transcriptome between Col-0 and autotetraploid (4 × Col-0) Arabidopsis. (**A**) Phenotypes of Col-0 (left) and 4 × Col-0 Arabidopsis (right) grown for 25 days on soil after stratification. (**B**) Flowering times of Col-0 and 4 × Col-0. Error bars represent means ± SEM (standard error of mean, *n* = 3 biological replicates). Statistical significance was analyzed by *t*-test, ***p < 0.001. (**C**) Phenotypes of rosette leaves of Col-0 and 4 × Col-0 Arabidopsis during vegetative stage. (**D**) Rosette leaf numbers of Col-0 and 4 × Col-0. Error bars represent means ± SEM (*n* = 3 biological replicates). Statistical significance was analyzed by *t*-test, ***p < 0.001. (**E**) Volcano plot for statistical significance against gene fold change between Col-0 and 4 × Col-0. Each gene was marked as a dot. Blue dots represent up-regulated genes, red dots represent down-regulated genes and green dots represent the other genes. (**F**) Numbers of up-regulated and down-regulated genes (|log_2_fold change| > 1) in 4 × Col-0 compared to Col-0.The data from three biological replicates were combined.

The wild type (Col-0, *Columbia*) and autotetraploid Arabidopsis (4 × Col-0) were confirmed by flow cytometry (Supplementary Figure S1). Compared with Col-0, autotetraploid Arabidopsis exhibited enlarged leaves, flowers and whole plant size (Figure 1). It was known that autotetraploid has increased trichome branch numbers, enlarged cells, stomata and seed sizes (48–50), and also provided enhanced tolerances to high salinity (4) and Cu^2+^ stress (51). We found that 4 × Col-0 plants flower 5 days later under long day condition (Figure 1A and B), and have about two more rosette leaves during the vegetative stage than Col-0 (Figure 1C and D).

We then examined the transcriptomes of 10 day Col-0 and 4 × Col-0 seedlings by RNA-sequencing. Transcriptome size-based normalization was performed to calculate the relative gene expression levels between Col-0 and 4 × Col-0. Compared with Col-0, we identified 436 up-regulated genes and 307 down-regulated genes (cutoff threshold, 2-fold) in autotetraploid (Figure 1E–F and Supplementary Table S1). Gene ontology (GO) analysis revealed that these differentially expressed genes associate with responses to starvation, metabolic process and stimuli (Supplementary Table S2).

### Increased inter-chromosome and decreased intra-chromosome arm interactions in autotetraploid Arabidopsis

**Figure 2.**
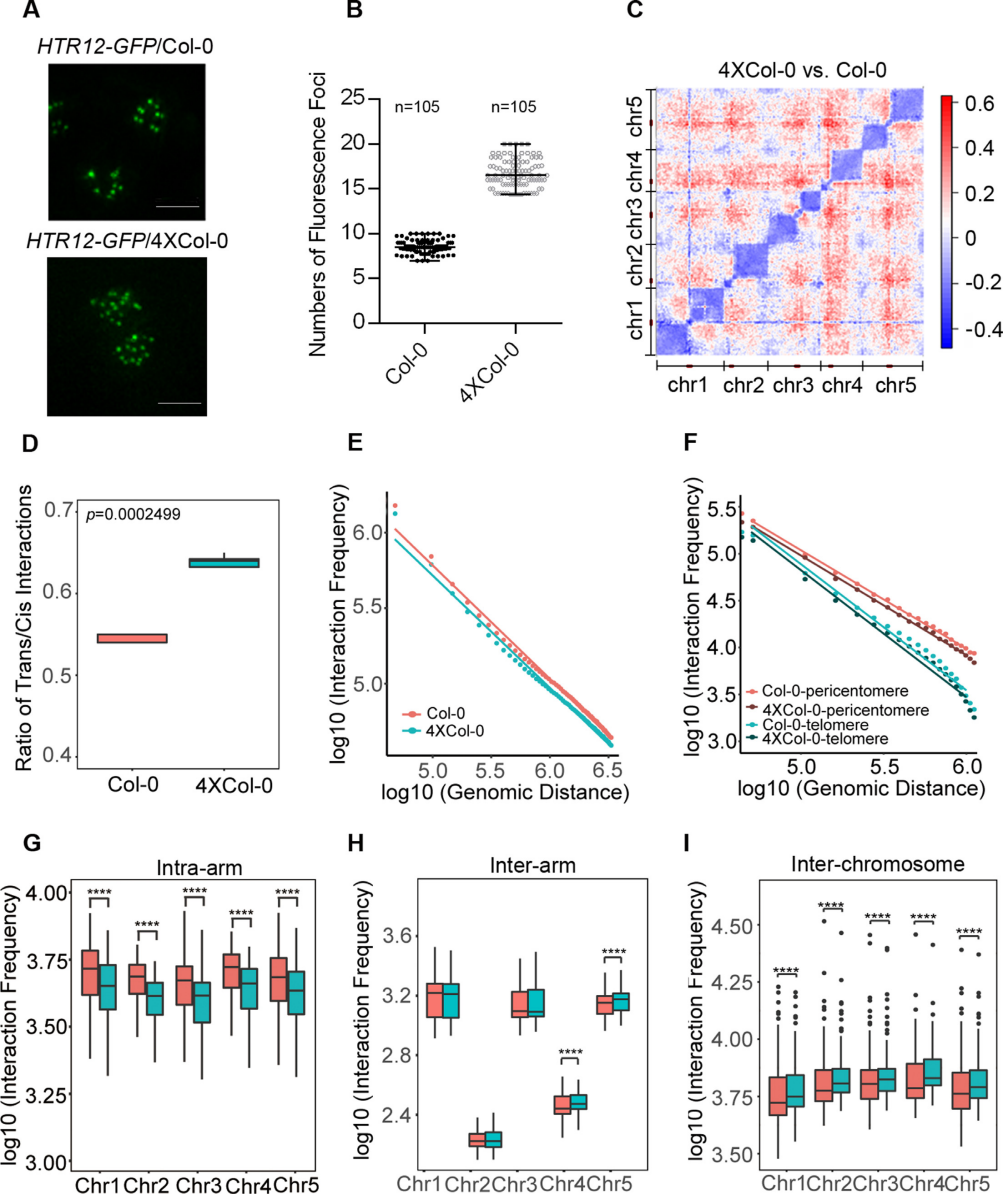
Comparison of 3D chromatin interaction profiles between Col-0 and 4 × Col-0 Arabidopsis. (**A**) *HTR12-GFP* labeled centromeres in guard cells of Col-0 and 4 × Col-0. Scale bar = 5 μm. (**B**) The numbers of centromeres in the guard cell nuclei of Col-0 and 4 × Col-0. The data were calculated by Imaris software from 105 Col-0 and 4 × Col-0 guard cell nuclei. (**C**) Differential chromatin interaction heatmap between Col-0 and 4 × Col-0 at 50 kb resolution. Chromosomes stacked from bottom left to up right were chr1, chr2, chr3, chr4 and chr5. The pericentromere of each chromosome was labeled in dark red bar. (**D**) Ratios of trans/cis interaction frequency were calculated across the whole genome in Col-0 (brick red) and 4 × Col-0 (blue). The *p* value was tested with Wilcoxon rank sum test. (**E**) Interaction decay exponents along with genomic distance at 50-kb resolution of Col-0 (brick red) and 4×Col-0 (blue). (**F**) Interaction decay exponents along pericentromeric and telomeric regions (5% of the ends) of 4 × Col-0 and Col-0. (**G**) Boxplots showing the intra-chromosome arm interaction frequencies between Col-0 (brick red) and 4 × Col-0 (blue). ****p < 3.83e–09, the data was tested by Wilcoxon's rank sum test. (**H**) Boxplots showing inter-chromosome arm interaction frequencies between Col-0 (brick red) and 4 × Col-0 (blue). ****p < 5.05e–05 (Wilcoxon's rank sum test). (**I**) Boxplots showing inter-chromosome interaction frequencies between Col-0 (brick red) and 4 × Col-0 (blue). ****p < 2.79e–05 (Wilcoxon's rank sum test).

To elucidate the effect of genome doubling on chromosome arrangement in living Arabidopsis plants, we transformed Col-0 and 4 × Col-0 Arabidopsis with *HTR12-GFP*, a centromere-specific histone H3 marker, using flower dipping (52,53). Transgenic plants expressing HTR12-GFP showed no obvious phenotypic changes compared to their corresponding non-transgenic lines, which indicated that the plants expressing this fusion protein have functional centromeres and normal cell cycles. For the analysis of centromere organization, three independent transgenic lines were used to determine the centromere distribution patterns in Col-0 and 4 × Col-0 Arabidopsis plants expressing HTR12-GFP. Small and bright HTR12 fluorescence foci could be detected in nuclei (Figure 2A). In wild type Arabidopsis, a range of 8–10 bright spots (with average number 8.5) were usually detected in each nucleus of guard cells, and ∼16–20 spots (with average number 16.7) were visualized in one of the paired tetraploid guard cells (Figure 2B). These results showed that the average number of centromere fluorescence foci in an autotetraploid nucleus were almost twice as much as those in diploid, indicating that centromeres in autotetraploid did not merge with each other after chromosome doubling and each chromosome territory (CT) (54) might be relatively independent.

To better understand the chromosomal architecture of autotetraploid Arabidopsis, we applied a Hi-C approach to compare the spatial organization of genomes in diploid and autotetraploid seedlings. After filtering and alignment to Col-0 reference genome (TAIR10), we obtained total 22 million and 32 million valid Hi-C reads from wild type and autotetraploid Arabidopsis, respectively (Supplementary Table S3), with a high reproducibility between the biological replicates (Supplementary Figure S2A and B). Differential analysis between Hi-C replicates of Col-0 or 4 × Col-0 was also performed to assess the stochastic variation of the experiments, the results showed no obvious interaction differences between the replicates of Col-0 or 4 × Col-0 (Supplementary Figure S2C and D).

We then calculated the interaction difference matrix from the reads with the same sequencing depth between Col-0 and 4 × Col-0 to reveal the chromosome clustering traits caused by genomic doubling. Genome-wide chromatin interaction difference matrix revealed that autotetraploid Arabidopsis has increased inter-chromosomal interactions, and reduced intra-arm interactions compared to Col-0 (Figure 2C). The ratio of trans/cis interaction confirmed this phenomenon at 50 kb binning resolution (Figure 2D), in which autotetraploid Arabidopsis showed a high ratio of trans/cis (inter-/intra) interaction frequency.

To better understand the distribution of intra-chromosomal contacts, we used Interaction Decay Exponents (IDEs) with power-law curves to reveal the difference of chromatin fiber characters between autotetraploid and diploid. The IDE could characterize chromatin packing by calculating a linear fit to the average interaction frequencies observed at given genomic distances. The results displayed that interaction frequency decreases as the genomic distance increases both in autotetraploid and wild type Arabidopsis. The intra-chromosomal interaction in autotetraploid showed decreased contact probabilities compared with Col-0 as a function of increasing genomic distance (Figure 2E). Beside, power-law curves of individual chromosome indicated that each chromosome showed the same interaction pattern with the whole genome in autotetraploid (Supplementary Figure S3). In addition, we observed the decreased intra-pericentromeric interactions (chromatin interactions within the pericentromeric region of a chromosome) and intra-telomeric interactions (chromatin interactions within a telomere of a chromosome) in autotetraploid (Figure 2F and Supplementary Figures S4 and S5). However, increased inter-pericentromeric interactions (chromatin interactions among pericentromeres of all chromosomes) and decreased inter-telomeric interactions (chromatin interactions among telomeres of all chromosomes) were observed in autotetraploid plants (Supplementary Figure S6A and B).

To better understand the chromatin packing in autotetraploid Arabidopsis, we compared the interaction frequencies within an arm of a chromosome (intra-arm), between two arms of a chromosome (inter-arm) or among different chromosomes (inter-chromosome). We found that the intra-arm interactions in 4 × Col-0 tend to have lower frequency compared with Col-0 (Figure 2G), and inter-arm interactions have no obvious interaction changes (Figure 2H), suggesting that autotetraploid Arabidopsis have a relative less compactness in chromosome arms compared to Col-0. In addition, the inter-chromosome interactions presented higher strengths in autotetraploid than those in Col-0 according to the interaction frequencies between a specific chromosome and the remaining chromosomes (brick red and blue boxplots in Figure 2I). We also found that the increased inter-chromosomal interactions for each chromosome are distributed largely uniformly across all other chromosomes (Supplementary Figure S6C).

In diploid Arabidopsis, a nuclear heterochromatic island structure was identified and known as KNOT (29,30). We found that autotetraploid also has the KEEs (Knot Engaged Elements)/IHIs (Interactive heterochromatic islands) regions in all chromosomes with slight weaker chromosome interaction frequency among them and flanking regions (Supplementary Figure S6D).

In order to understand the spatial chromosome arrangement of the duplicated genome in nucleus, we calculated the log-ratio of the observed inter-chromosome interaction frequencies and the expected interaction frequencies as described (55). The matrix showed that autotetraploid has equal interactions among all chromosomes to those of Col-0, indicating that genome duplication has a limited effect on the spatial interactions of chromosome territories (Supplementary Figure S6E).

### Specific chromatin structure domains were changed during autopolyploidization

**Figure 3.**
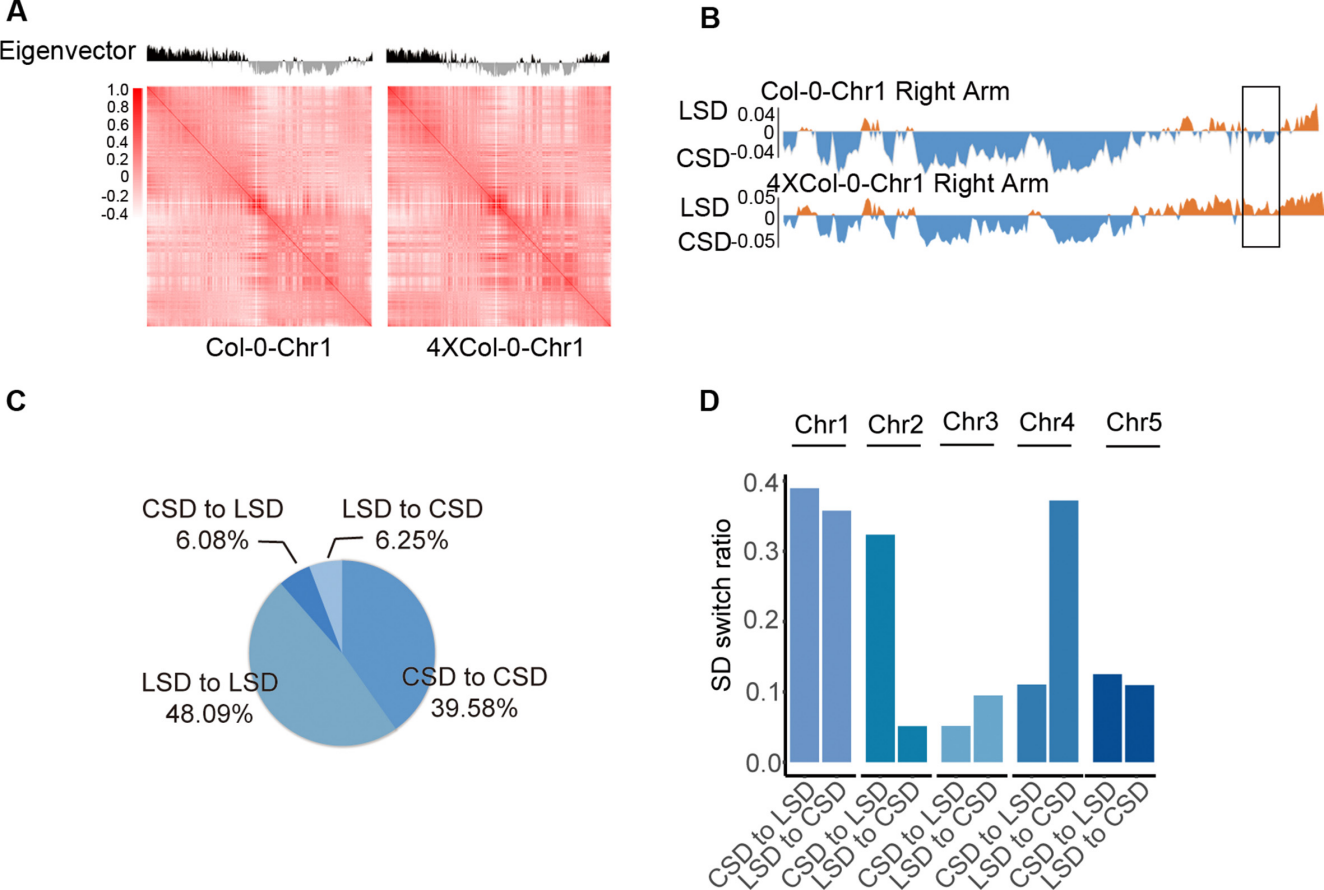
Comparison of chromatin structure domains between Col-0 and 4 × Col-0 Arabidopsis. (**A**) Pearson correlation coefficient matrix and its respective first eigenvector of chr1. The dark regions with positive values in the first eigenvector represent loose structure domains (LSD) and the gray regions with negative values represent compact structure domains (CSD). Genomic bin size: 50 kb. (**B**) First eigenvector of chr1 right arm (18 000 000–30 427 671 bp). CSDs are presented in blue and LSDs in orange. The dark block highlights the transition region from CSD in Col-0 to LSD in 4 × Col-0 (28 850 000–29 550 000 bp). (**C**) Pie chart representing the percentages of genomic structure domain changes between Col-0 and 4 × Col-0. LSD to LSD represents the loose chromatin domains in both Col-0 and 4 × Col-0. CSD to CSD represents the compact chromatin domains in both Col-0 and 4 × Col-0. LSD to CSD denotes loose chromatin domains in Col-0 but compact in 4 × Col-0, whiles CSD to LSD denotes compact chromatin domains in Col-0 but loose in 4 × Col-0. (**D**) Bar graph showing the statistics of structure domain changes in all chromosomes between Col-0 and 4 × Col-0. The annotations of CSD to LSD and LSD to CSD are the same as in (C).

Principal Component Analysis (PCA) of the distance-normalized and correlated intra-chromosomal interactions (17,29) was previously used to determine the arrangements of discrete structure domains. To determine whether there are any differences in the chromatin structure domain organization between Col-0 and 4 × Col-0, we compared the structure domains through the first principal component at 50 kb resolution. We divided Arabidopsis diploid and autotetraploid genomes into two types of distinct structure domains, one for the compacted structure domains (CSD) with negative values in the first eigenvector and the other for the loose structure domains (LSD) with positive values in the first eigenvector (Figure 3A and Supplementary Figure S7) (29). Our results indicated that the autotetraploid and diploid have similar interaction matrix patterns of the discrete structure domains (Figure 3A and Supplementary Figure S7), with certain regions showing changes in structure domains from CSD to LSD or LSD to CSD (Figure 3B). Compared with Col-0, 48.09% and 39.5% of the genome showed conserved LSD and CSD in autotetraploid Arabidopsis (Figure 3C), respectively. Interestingly, we found that 6.08% of structure domains in Col-0 genome converted from CSD to LSD, and 6.25% converted from LSD to CSD in autotetraploid (Figure 3C). To better characterize SD switching, we quantified the chromosome preferences. We found that the compartment transitions from LSD to CSD occur mostly on chromosome 1 (Chr1) and Chr4, and CSD to LSD mainly on Chr1 and Chr2 (Figure 3D). Quantification of chromatin interactions in LSD and CSD revealed that there is a higher interaction frequency in CSD and lower in LSD both in 4 × Col-0 and Col-0 (Supplementary Figure S7E).

We also analyzed the genome elements in the chromosome regions converted from CSD to LSD with RepeatMasker. The results showed that the CSD to LSD regions contain retroelements (1.89%), DNA transposons (1.36%), small RNA (0.08%), satellite DNA (0.06%), low complexity DNA (0.41%) and simple repeat sequence (1.29%). Then we annotated the genes in the CSD to LSD switched regions with ARAPORT (Arabidopsis Information Portal). We found that there were 1749 coding genes (83%), 310 non-coding genes (14.7%) and 48 pseudogenes (2.3%).

Similarly, we found that LSD to CSD regions contain retroelements (3.75%) and DNA transposons (2.51%), small RNAs (0.14%), low complexity DNA (0.38%) and simple repeat sequence (1.29%). The same gene annotation was also performed in these regions. The results showed that there were 939 coding genes (83.3%), 160 non-coding genes (14.2%) and 28 (2.5%) pseudogenes.

### The specific chromatin structure domain correlates with histone modifications H3K4me3 and H3K27me3 in autotetraploid.

**Figure 4.**
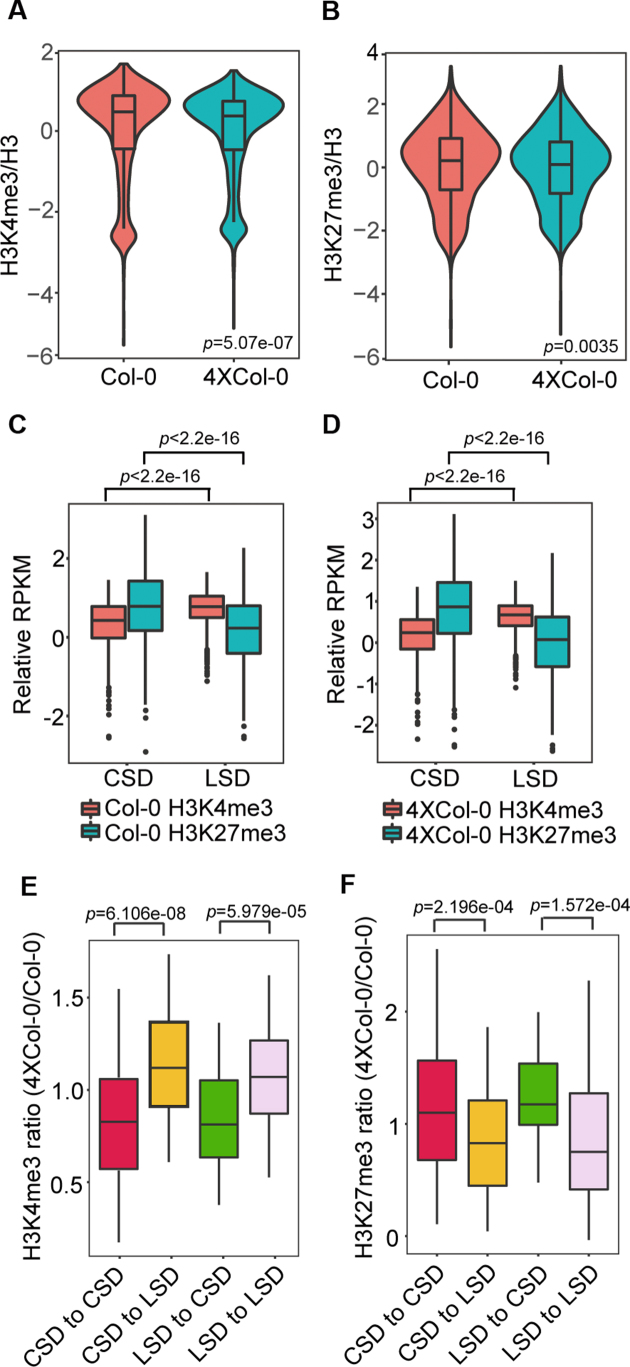
The relationship between chromatin status and histone modifications in Col-0 and 4 × Col-0 Arabidopsis. (**A**) H3K4me3 levels in genomes of Col-0 and 4 × Col-0 Arabidopsis, plotted as the ratio of H3K4me3 to H3 ChIP-seq. (**B**) H3K27me3 levels in genomes of Col-0 and 4 × Col-0 Arabidopsis, plotted as the ratio of H3K27me3 to H3 ChIP-seq. (C, D) H3K4me3 and H3K27me3 levels in CSD and LSD in Col-0 (**C**) and 4 × Col-0 (**D**). CSD regions have a higher level of H3K27me3 RPKM (reads per kilobase per million mapped reads) and lower level of H3K4me3 RPKM in both Col-0 and 4 × Col-0, whereas LSD regions have a higher level of H3K4me3 RPKM and lower level of H3K27me3. (**E**) Boxplot showing the ratio of H3K4me3 in 4 × Col-0 to Col-0 at the converted or no-converted structure domains. (**F**) Boxplot showing the ratio of H3K27me3 in 4 × Col-0 to Col-0 at the converted or no-converted structure domains. The *p* values were tested by Wilcoxon–Mann–Whitney test.

The changes of chromatin structure domains in the autotetraploid prompted us to test whether histone modifications associate with these alterations. To examine the epigenomic dynamics between autotetraploid and diploid Arabidopsis, we analyzed H3K4me3 and H3K27me3 histone modifications by ChIP-seq. At the genome-wide scale, we found that the levels of H3K4me3 and H3K27me3 in 4 × Col-0 are only slightly lower than those in Col-0 (Figure 4A and B) with high Pearson's correlation coefficients between the two replicates of each experiment (Supplementary Figure S8). However, there were no obvious differences of H3K4me3 and H3K27me3 on each individual chromosome (Supplementary Figure S9A and B). These results suggested that the global levels of H3K4me3 and H3K27me3 were not obviously affected by the genome duplication. We then analyzed the H3K4me3 and H3K27me3 modifications on chromatin structure domains. The results showed that the CSD compartments in both Col-0 and 4 × Col-0 had a higher level of H3K27me3 and lower level of H3K4me3 compared to LSD (Figure 4C and D), and LSD compartments in both Col-0 and 4 × Col-0 had a lower level of H3K27me3 and higher level of H3K4me3 compared to CSD (Figure 4C and D).

To gain insight into the correlation between histone modification and chromatin domain, we analyzed the distribution of histone modifications in the chromatin regions with converted and unconverted structure domains. Compared to the unconverted compacted structure domain (CSD to CSD in Figure 4E), we found that the level of H3K4me3 in 4 × Col-0 was higher in the chromatin regions where CSDs in Col-0 were converted to LSDs in 4 × Col-0 (CSD to LSD in Figure 4E and Supplementary Figure S9C). Similarly, compared with the unconverted loose structure domain (LSD to LSD in Figure 4E), we observed a lower level of H3K4me3 in 4 × Col-0 in the regions where LSDs in diploid were converted to CSDs in 4 × Col-0 (LSD to CSD in Figure 4E and Supplementary Figure S9C). Similarly, we compared the H3K27me3 modification level between converted and unconverted structure domains. The results showed that the level of H3K27me3 in 4 × Col-0 was lower in the CSD to LSD converted regions (Figure 4F and Supplementary Figure S9D) compared to unconverted CSD (CSD to CSD in Figure 4F), and the level of H3K27me3 in 4 × Col-0 was higher in the LSD to CSD converted regions (Figure 4F and Supplementary Figure S9D) compared to unconverted LSD (LSD to LSD in Figure 4F). We also checked the correlation between SD and the changes in H3K4me3 and H3K27me3 with Jaccard index. The results showed that the histone modifications correlate with the switched SDs (Supplementary Table S4). Together, these results indicated that the loose structure domains tend to enrich in the active histone modification H3K4me3 and the compacted structure domains are associated with the repressive histone modification H3K27me3. When the chromatin status in some loci changes upon genome duplication, the histone modifications in these domains altered accordingly.

We also analyzed the histone modification in the unconverted regions. Interestingly, we found that the CSD to CSD regions in the 4 × Col-0 had a higher H3K27me3 level than that of Col-0, and the LSD to LSD in the 4 × Col-0 had higher H3K4me3 level and lower H3K27me3 level than that of Col-0 (Supplementary Figure S9C and D), which implicated that the compacted chromatin regions might become more condensed and the loose chromatin regions become more decondensed after genome duplication.

### The transcriptions of a population of genes in autotetraploid Arabidopsis are specifically regulated by chromatin interaction

Differential expression analyses showed that the transcription levels of 743 genes were obviously changed in autotetraploid. First, we analyzed whether the changes in gene expressions were associated with histone modifications by comparing the H3K4me3 and H3K27me3 modification profiles between 4 × Col-0 and Col-0. The differentially-expressed genes did not show obvious differences in patterns and levels of H3K4me3 and H3K27me3 in autotetraploid Arabidopsis compared with these genes in wild type (Supplementary Figure S10A–D). Similar to all genes, the H3K4me3 was observed to be enriched in transcription-start site (TSS) (Supplementary Figure S10A to B) in both up- and down-regulated genes. Interestingly, compared to all genes, H3K27me3 shows elevated levels only in the bodies of down-regulated genes but not in up-regulated genes both in Col-0 and 4 × Col-0 (Supplementary Figure S10C to D). In addition, the relative amounts of H3K4me3 and H3K27me3 in differentially expressed genes were similar in Col-0 and 4 × Col-0 with slight down-regulation of H3K27me3 for up-regulated genes (Supplementary Figure S10E and F).

**Figure 5.**
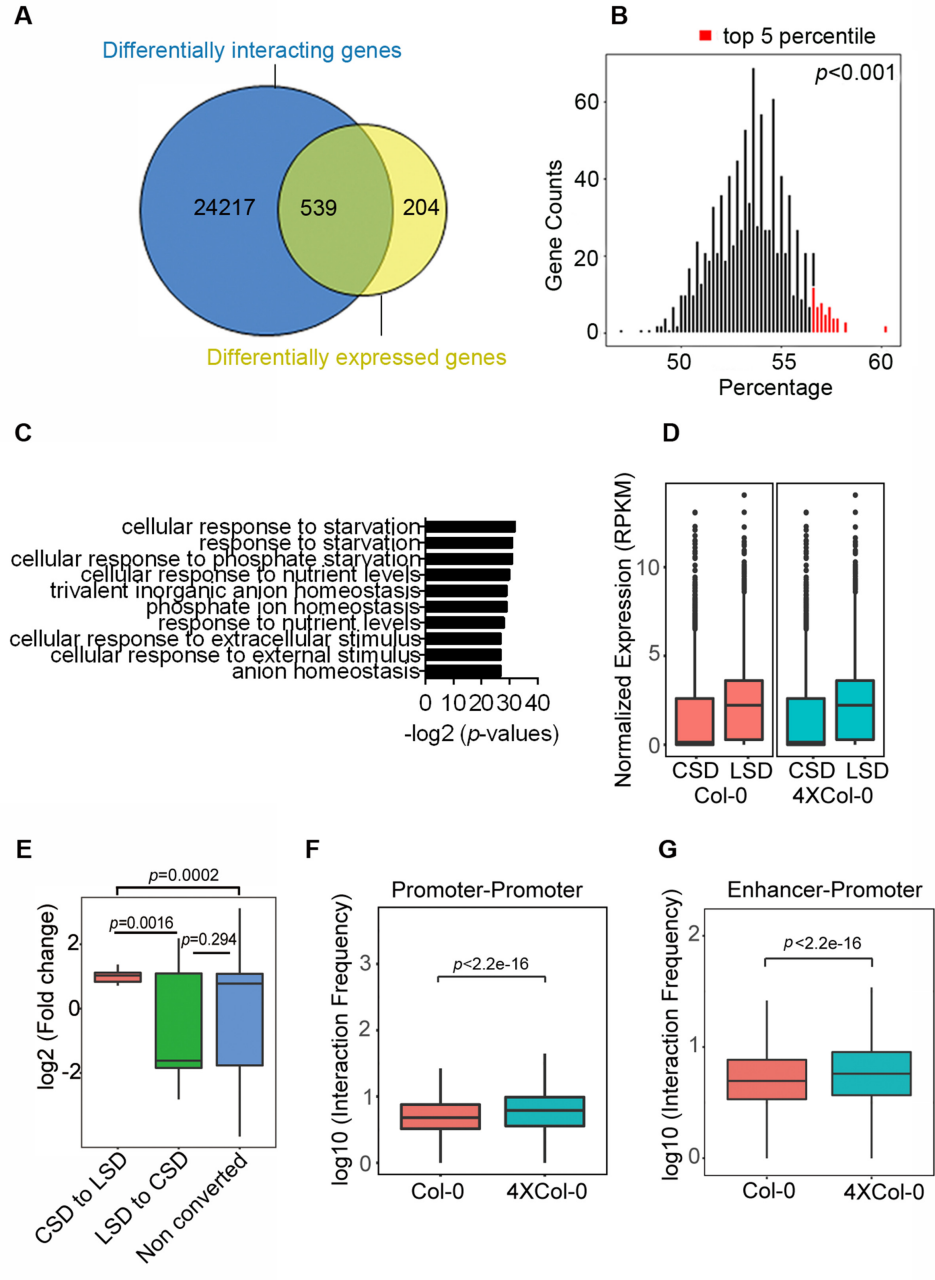
The relationship between chromatin structure and gene transcription in Col-0 and 4 × Col-0 Arabidopsis. (**A**) Venn diagrams showing numbers of genes in differential interaction bins (dark blue) and differentially expressed genes (yellow) in Col-0 and 4 × Col-0 Arabidopsis. The 539 genes were overlapped from the two conditions. (B) Histogram of randomly selected no-regulated genes in differentially interaction bins (*n* = 1000). The top 5 percentile of randomly selected control genes was labeled in red. (**C**) GO analysis of overlapped genes between the differentially expressed genes and those in differential interaction bins. (**D**) Expression levels of genes in the CSD (brick red) and LSD (blue) in Col-0 and 4 × Col-0 Arabidopsis. (**E**) Boxplot of the log2 fold change of expression levels from genes residing in the regions with chromatin structure domain transition between LSD and CSD or without domain transition (no converted). (**F**) Promoter-promoter interaction frequencies in Col-0 and 4 × Col-0 Arabidopsis. (**G**) Enhancer-promoter interaction frequencies in Col-0 and 4 × Col-0 Arabidopsis. The *p* values were tested by Wilcoxon rank-sum test.

To further address the mechanism underlying the gene regulation during genome duplication, we asked whether the observed differential gene expressions between autotetraploid and diploid associated with chromatin packing. We aligned the differentially expressed genes with the genes localized in the altered interaction frequency chromatin bins. We found that most of genes (539/743, about 72.5%) in the differentially expressed gene library localized in the differential interaction bins (Figure 5A and Supplementary Table S5). Bootstrapping randomized analysis was then performed to analyze the confidence of this phenomenon. To this end, we randomly selected 1000 group of equal number (743) of no-regulated genes to determine the percentage of those genes fallen into the differentially interacting bins. The result showed that the top 5 percentile of randomly selected control genes is <60%, which is lower than the percentage of the differentially expressed genes localized in the differential interaction bins (72.5%) (Figure 5B), indicating that the gene expression in autotetraploid Arabidopsis associated with chromatin interaction. We also found that these 539 genes were related to starvation and stimulus response pathways (Figure 5C), consistent to the GO analysis of the differentially expressed genes between diploid and autotetraploid (Supplementary Table S2).

To uncover the structure domains (SD) in the transcriptional regulation of the genome-doubled Arabidopsis, we analyzed the normalized RNA-seq RPKM in LSDs and CSDs, and found that genes in the LSD compartments display higher expression levels than those in CSD compartments for both Col-0 and 4 × Col-0 (Figure 5D). We then analyzed the differentially-expressed genes at the regions where the SD transitions occurred. We found that the differentially expressed genes in the regions of CSD to LSD tend to be obviously up-regulated (with 37 up-regulated genes and 7 down-regulated genes) (Figure 5E), and differentially expressed genes in the regions of LSD to CSD in autotetraploid (Figure 5E) tend to be slightly down-regulated (with 16 down-regulated genes and 13 up-regulated genes). We also found that a number of differentially expressed genes (657 genes) localize in the un-converted SD regions (Figure 5E), indicating that the chromatin interaction, but not the SD transition, is the major factor for the gene regulation upon genome duplication in Arabidopsis.

To gain more insight into the transcription regulation, we tested the genome-wide promoter-mediated interactions to address possible mechanisms through long-range interactions. We aligned all the promoters into the interaction bins and calculated the numbers of interactions among these bins. The results indicated that the promoter-promoter interaction frequency in the autotetraploid was higher than that in wild type (Figure 5F), implicating that the promoters in the polyploid have more intense interactions among them. Next we examined the interaction frequencies of other cis-regulatory elements like enhancers using the candidate enhancer library identified from DNase I hypersensitive site-sequencing data (46). We found that the enhancer-promoter interaction frequency in autotetraploid is also higher than that in diploid Arabidopsis (Figure 5G), implicating that long-range chromatin interaction might play an important role in the regulation of the doubled genome.

### Arabidopsis genome duplication remodeled the *FLC* gene loop

**Figure 6.**
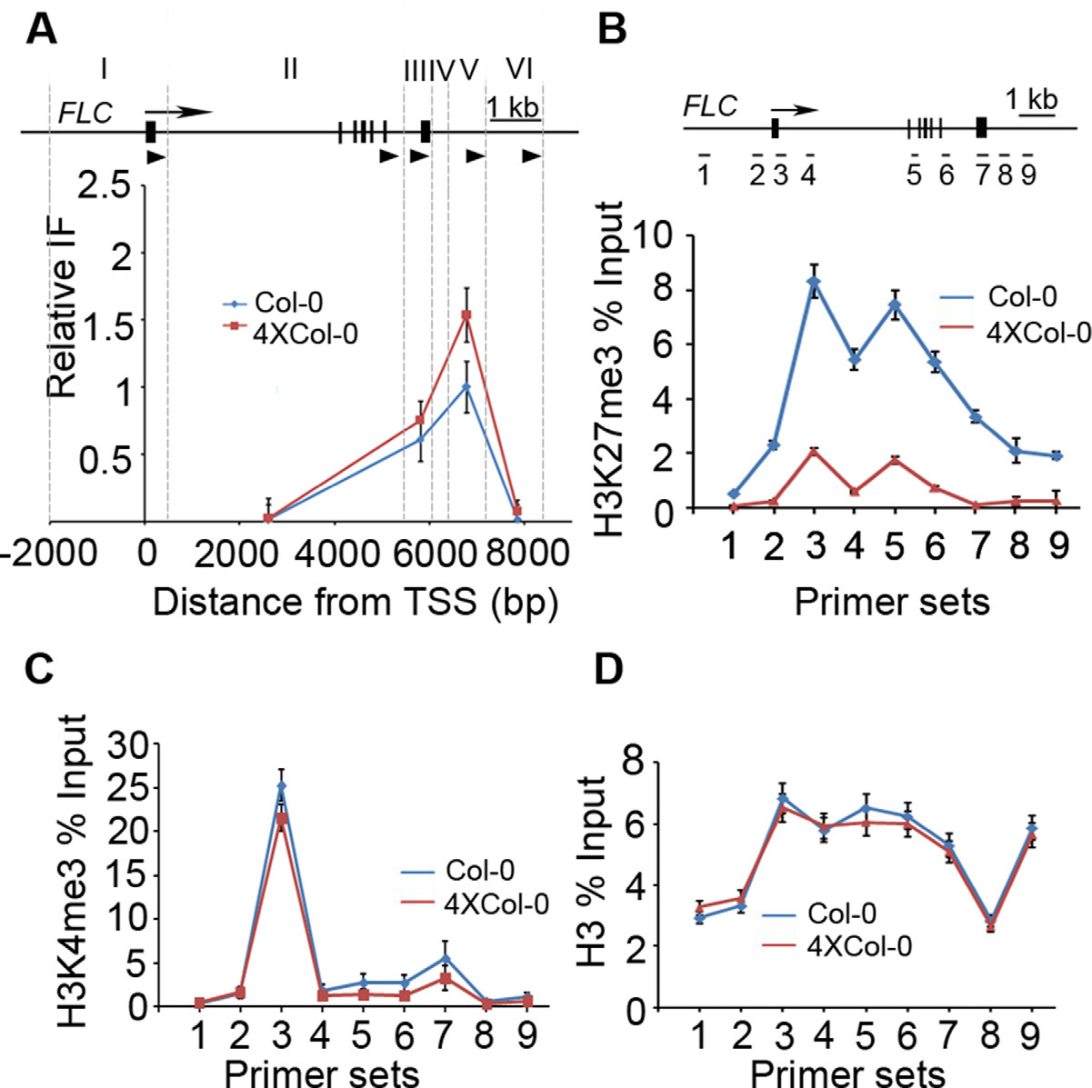
Comparisons of gene loop and histone modifications in *FLC* locus between Col-0 and 4 × Col-0 Arabidopsis. (**A**) Chromatin interaction frequency of *FLC* in wild type and autotetraploid as revealed by 3C. A schematic representation of the *FLC* locus is shown at the top, and the arrow indicates the position of the transcription start site. Exons are represented as black boxes and introns as black lines, dotted lines indicate *Bam*H1 or *Bg*lII restriction sites, and the primer locations were marked by black triangles. Roman numbers represent the *FLC* gene regions. IF means interaction frequency. (B–D) ChIP-PCR assays to analyze the H3K27me3 (**B**), H3K4me3 (**C**) and H3 (**D**) enrichments at *FLC* locus. A schematic representation of the *FLC* locus is presented at the top of (B). The positions of nine primer pairs (1–9) used for ChIP-quantitative PCR are indicated. Values came from three biological replicates each with three technical repeats, and were presented as ratio to 1% Input.

It was reported that autotetraploid confers a series of physiological consequences upon genome duplication. An obvious phenotype of the autotetraploid Arabidopsis plants is later flowering which might be regulated by the differentially expressed genes. We noticed that the transcript level of a floral repressor gene *FLOWERING LOCUS C* (*FLC*) was higher in autotetraploid than that in Col-0 in RNA-seq data (Supplementary Table S1). Recent studies revealed that the N- and C-terminal flanking regions of *FLC* form a gene loop which is affected by the chromatin-remodeling factor BAF60 (47,56,57). To compare the gene loop in *FLC* locus between autotetraploid and Col-0, we performed Chromatin Conformation Capture (3C) experiments. The results showed that the relative amount of *FLC* loop increased in autotetraploid plants (Figure 6A).

We then assessed the levels of H3K27me3 and H3K4me3 by chromatin immunoprecipitation (ChIP-PCR) using primers covering promoter, exon and intron of *FLC* (Supplementary Table S6) (57). We found that the level of H3K27me3 in the autotetraploid decreased across the full length of gene locus compared with Col-0 (Figure 6B and C). In contrast, we observed no changes in the levels of H3K4me3 and H3 which was served as a control (Figure 6D). The elevated levels of gene loop and the loss of H3K27me3 modification in *FLC* locus in autotetraploid implicated the role of chromatin status in the flowering regulation.

## DISCUSSION

Polyploid with two or more sets of genomes is more common in plants. Most researches focused on the role of polyploid in eukaryote evolution (5,58,59). Comparing with allopolyploid, autopolyploid having homologous genomes received less attention. In fact, the rate of autopolyploid formation is higher than allopolyploid in nature (32). Our results showed that genome-doubled Arabidopsis acquires morphologic traits like, more and larger rosette leaves and elevated biomass which are similar to other polyploidy plants (31,60). We also noticed that the genes related to physiological and ecological tolerances were changed in autotetraploid, which might relate to the phenotypes of autotetraploid with environmentally adaptive benefits.

In this work, we investigated how genome doubling affects the chromatin architecture and the relationship between chromatin structure and transcription regulation. Recent studies have illustrated that polyploidy genome is dynamic and undergoes structural alterations (61–63). The separated centromeres marked by Arabidopsis *CenH3* (*HTR12)-GFP* in 4 × Col-0 indicated that the homologous chromosomes do not merged together after genome duplication (Figure 2A), implicating that each chromosome occupies a relatively separated space. Also, our results showed that the ratio of observed and expected chromosome interaction frequency was similar in wild type and autotetraploid Arabidopsis, which suggested that each chromosome forms a relatively independent chromosome territory in autotetraploid (Supplementary Figure S3A).

Our comparative analysis of the genome-wide chromatin interaction between 4 × Col-0 and Col-0 by Hi-C revealed that autotetraploid Arabidopsis has its specific conformation with decreased intra-arm interactions and increased inter-chromosome interactions (Figure 2). It was reported that the volume of autotetraploid nuclei increased approximately only by a factor of 1.7 but not 2 compared with diploid, the relatively smaller nuclear volume indicated the higher average chromatin density (64). Thus, one postulation is that the increased inter-chromosome interactions in autotetraploid lead to higher chromosomal compaction, resulting in a smaller nuclear size.

We found that the genomes of autotetraploid and diploid showed similar compacted structure domains (CSDs) and loose structure domains (LSDs) with ∼12% structural domains switched between CSD and LSD (Figure 3C), indicating that genome duplication can remodel the chromatin structures in some loci. Analysis of switched domains showed that the majority of transition events occur on chr1, chr2 and chr4. We proposed that the big and gene-rich chromosome 1 might have a higher intensity of intra-chromosome communication. Previous studies reported that the entire left arm of Arabidopsis chromosome 4, including pericentromere regions and NAD (nucleolus associated chromatin domains), associates with nucleolus, and the *nucleolus organization region 4* (*NOR4*) is actively transcribed. In contrast, the short arm of chromosome 2 is excluded from nucleolus and *NOR2* is inactive (65,66). In autotetraploid, major chromatin domain switches of CSD to LSD occur on chr2, which might result from the relocation of chr2 to nucleolus and decondensation of the chromatin, resulting in the active transcription. For chr4, the chromatin domain switches of LSD to CSD might implicate that *NOR4* is not associated with nucleolus in autotetraploid, and the transcription of *NOR4* rRNA or NAD-related genes failed to be intensely activated. Further studies are necessary to test these hypotheses.

Histone modifications of H3K4me3 and H3K36me3 on euchromatin were known to correlate to LSDs, whereas the polycomb-associated H3K27me3 is connected with heterochromatin that contains CSDs (17,25,29). In contrast, H3K27me1, H3K18ac and H3 showed weak correlations with the chromatin packing status (29). We assessed the relationships between H3K27me3/H3K4me3 and chromatin status in autotetraploid and wild type. Our results showed that the global H3K4me3 and H3K27me3 did not show obvious difference after genome doubling, which might be due to that autotetraploid has two identical genomes. In addition, we found that LSDs (loose structure domains) tends to have a higher level of the active marker (H3K4me3) and a lower level of repressed marker (H3K27me3), whereas CSDs have a lower level of H3K4me3 and higher level of H3K27me3 in both Col-0 and genome-duplicated Col-0. Importantly, we found that H3K27me3 and H3K4me3 could reflect the genome structure domain transformation. The CSD to LSD converted regions in the autotetraploid present a higher level of H3K4me3 and lower level of H3K27me3, while the LSD to CSD converted regions showed contrary tendency (Figure 4E, F and Supplementary Figure S9C and D), indicating that chromatin structure domain switches are correlated with the changes of histone modifications upon genome duplication. It was known that H3K4me3 is an active marker while H3K27me3 a repressive marker of gene expression (67). In autotetraploid Arabidopsis, we found that the H3K4me3 profile did not show obvious differences both in up- or down-regulated genes compared with the total genes (Supplementary S10A, B). However, although the modification pattern of H3K27me3 in the up- and down-regulated genes were similar both in Col-0 and 4 × Col-0, the up-regulated genes have lower levels of H3K27me3 while the down-regulated genes have higher levels of H3K27me3 compared with the total genes (Supplementary S10C, D), implicating that H3K27me3 might play a role in defining chromatin identity for the transcriptional regulation of the related genes during genome doubling. Further quantification analysis showed that the total level of H3K4me3 or H3K27me3 in up- or down- regulated genes did not reveal obvious differences between Col-0 and 4 × Col-0 (Supplementary Figures S10E and F), suggesting that the transcription changes in autotetraploid Arabidopsis are not tightly linked to the H3K4me3 and H3K27me3 modifications, similar to the previous study that changes in H3K27me3 were not significantly correlated with gene expression at shoot apical meristems during the transition to flowering in Arabidopsis (68).

To better understand the gene regulation underlying genome duplication in the autotetraploid, we addressed the relationship between chromosome structure and gene transcription. Our results showed that 72.5% of the differentially expressed genes localized in the differential interaction bins, which indicated that the chromatin interaction might play a role in the regulation of the doubled genome. In addition, we found that a large amount of these differentially expressed genes associate with and responses to stimuli, suggesting that the chromatin interaction might be important for the plant to acclimation (Figure 5C). Although some differentially expressed genes localize to the converted structure domains where the genes tend to be up-regulated in the CSD to LSD regions, most of the differentially expressed genes localize in the unconverted regions, which implied that the SD transition is not a major factor in gene regulation upon genome duplication. According to these results, we propose that the chromatin interaction is important in regulating gene expression in autotetraploid.

At the specific gene locus of *FLC*, we found that there are higher intensities of loops around *FLC* gene in autotetraploid Arabidopsis compared with diploid. This structure might help to recruit more RNA polymerase complexes to activate the *FLC* expression to repress flowering. The elevated promoter-promoter and enhancer-promoter interaction frequencies in autotetraploid confirmed the elevated level of long-range interactions in autotetraploid. We also found that the level of H3K27me3 modification but not H3K4me3 decreased on *FLC* gene in autotetraploid Arabidopsis, which promoted the expression of *FLC* in another way. Together, our results suggested that H3K4me3 and H3K27me3 modifications are not the major players in the global regulation of autotetraploid genomes. For some specific loci, they may synergistically regulate gene expression together with chromatin structure in autotetraploid. Our work thus revealed a new perspective regarding the genome organization and gene regulation in autotetraploid plants.

## DATA AVAILABILITY

The raw and processed Hi-C, ChIP-seq and RNA-seq datasets have been submitted to NCBI Gene Expression Omnibus (GSE114950).

## Supplementary Material

gkz511_Supplemental_Files
