# Supplementary material for: The effects of Arabidopsis genome duplication on the chromatin organization and transcriptional regulation
Source: Nucleic Acids Res. 2019 Jun 11;47(15):7857–69. doi: 10.1093/nar/gkz511 (PMC6736098; doi:10.1093/nar/gkz511)
Supplement: gkz511_Supplemental_Files [file gkz511_supplemental_files.zip › Supplementary Table 6 The primers used in this work.docx]

**The primers used in this work**

| Primers | Sequence | Purpose |
| --- | --- | --- |
| FLC 1 F | GACATATCCAGAAAAGGGCAAG | Chip-qPCR |
| FLC 1 R | GGTGAATGTACGGCATGATTT | Chip-qPCR |
| FLC 2 F | ATCCGTATCGTAGGGGAGGAAAGA | Chip-qPCR |
| FLC 2 R | GAAGACAAGATTGCCACGTGTACC | Chip-qPCR |
| FLC 3 F | AAACGTCGCAACGGTCTCAT | Chip-qPCR |
| FLC 3 R | AAACCCAGGTAAGGAAAAGGCG | Chip-qPCR |
| FLC 4 F | CACAGTAGTTTTGAATTTTGGTAGCTT | Chip-qPCR |
| FLC 4 R | TGAAGTAGCATATGTGCGGTAAG | Chip-qPCR |
| FLC 5 F | CGATATGGGAAACAGCATGCTGA | Chip-qPCR |
| FLC 5 R | GGGCTATGAAAATTGCGGTATGC | Chip-qPCR |
| FLC 6 F | CAGGTTTTGGCTAGCCAGGTAA | Chip-qPCR |
| FLC 6 R | CACACAACACGCAGTGCTTA | Chip-qPCR |
| FLC 7 F | GTGGGAGCAGAAGCTGAGAT | Chip-qPCR |
| FLC 7 R | GGAGAGTCACCGGAAGATTG | Chip-qPCR |
| FLC 8 F | TTAAAGTCATTGCTGTTAACGTGAC | Chip-qPCR |
| FLC 8 R | TGACGGAACTACGGAAGTGA | Chip-qPCR |
| FLC 9 F | AAGGCAACACAAACTTTCTCG | Chip-qPCR |
| FLC 9 R | TGTCGGCTGGATCCACTT | Chip-qPCR |
| 3C_Ib | CGTGCTCGATGTTGTTGAGT | 3C |
| 3C_IId | TTGGTTTCCTTGAAGGTTGTG | 3C |
| 3C_IIId | TGGCCAAGAGACTTTGTGTG | 3C |
| 3C_IVd | CTTCCGTAGTTCCGTCATCC | 3C |
| 3C_Vd | AAGGCAACACAAACTTTCTCG | 3C |
| 3C_VId | TGTCCTTGGCAAGAAAGAGG | 3C |
| LC_F | TTCATTTAGCAACGAAAGTGAAAAC | 3C |
| LC_R | TTGTGTTTTGAAGACAAGATTGC | 3C |
| UBC_QPCR_F | CTGCGACTCAGGGAATCTTCTAA | 3C |
| UBC_QPCR_R | TTGTGCCATTGAATTGAACCC | 3C |

A
