# Supplementary material for: The effects of Arabidopsis genome duplication on the chromatin organization and transcriptional regulation
Source: Nucleic Acids Res. 2019 Jun 11;47(15):7857–69. doi: 10.1093/nar/gkz511 (PMC6736098; doi:10.1093/nar/gkz511)
Supplement: gkz511_Supplemental_Files [file gkz511_supplemental_files.zip › Supplementary data.pdf]

Supplementary Figure 1

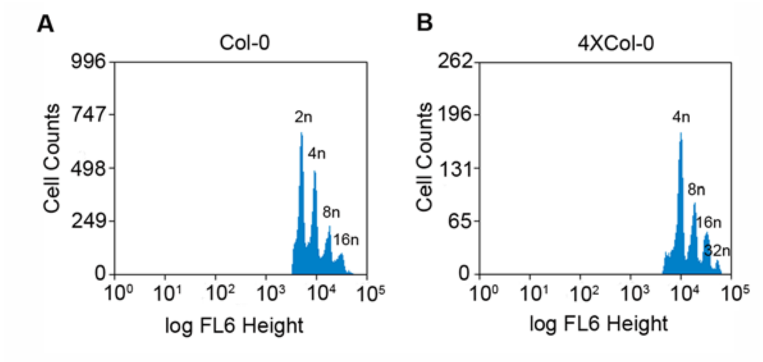

Supplementary Figure 1. Flow-Cytometric DNA histograms for wild type and autotetraploid Arabidopsis. (**A**) The DNA histogram of wild type (Col-0) Arabidopsis, the endoreduplicated cells were labeled as 4n, 8n, 16n. (**B**) The DNA histogram of autotetraploid (4×Col-0) Arabidopsis, the endoreduplicated cells were labeled as 8n, 16n and 32n. The nuclei were stained with DAPI, and FL6 means the channel of DAPI.

Supplementary Figure 2

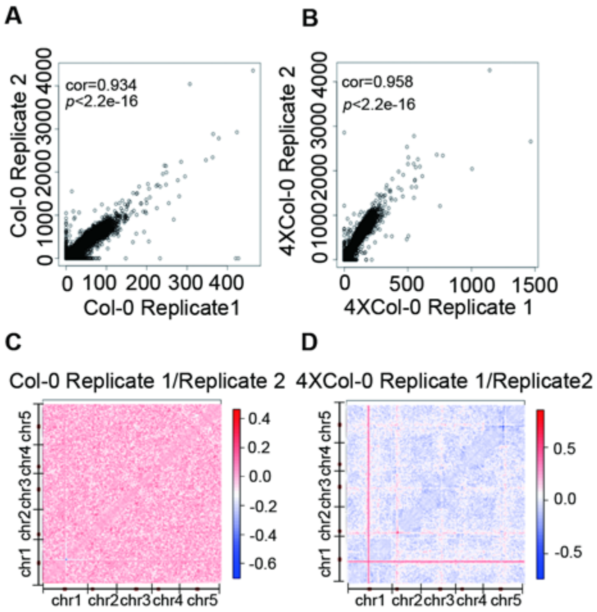

Supplementary Figure 2. Reproducibility analysis of Hi-C biological replicates. (**A**) Scatter plot of Col-0 Hi-C replicates. Numbers on the X- and Y- axes mean the interaction frequencies in 50 kb bins from Col-0 replicates. Pearson correlation was calculated according to these frequencies. (**B**) Scatter plot of 4×Col-0 Hi-C replicates. Numbers on the X- and Y- axes mean the interaction frequencies in 50 kb bins from 4 ×Col-0 replicates. Pearson correlation was calculated according to these frequencies. (**C**) The differential interaction heatmap between Col-0 replicate 1 and replicate 2 at 50 kb resolution. (**D**) The differential interaction heatmap between 4×Col-0 replicate 1 and replicate 2 at 50 kb resolution.

Supplementary Figure 3

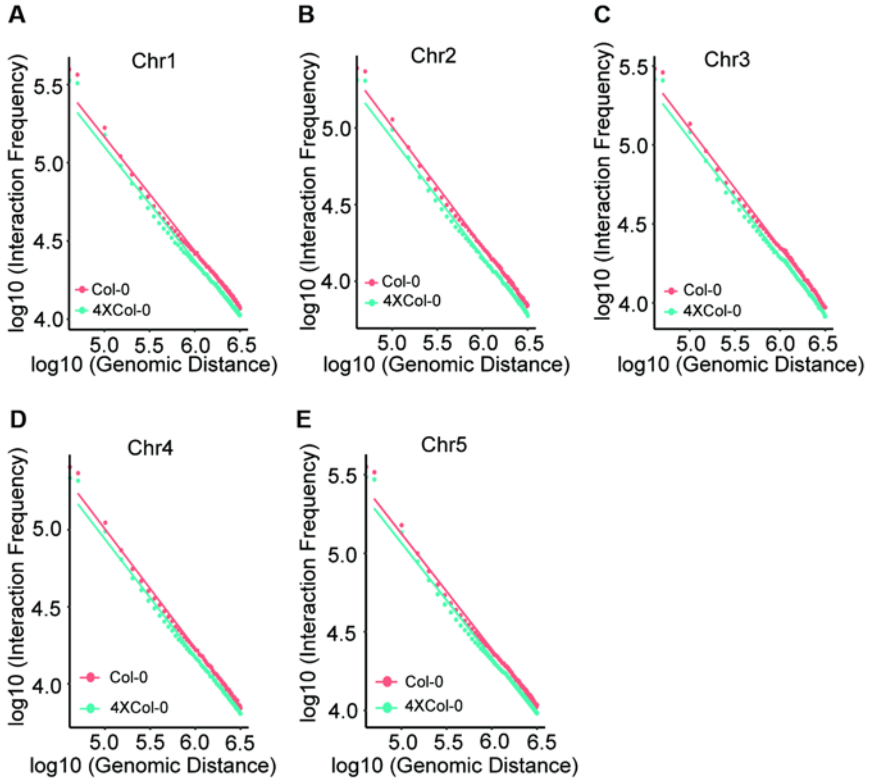

Supplementary Figure 3. Interaction decay exponents of Col-0 and 4×Col-0 chromatin interactions. (**A-E**) Interaction decay exponents of chr1, chr2, chr3, chr4 and chr5, respectively. The interaction frequency in different chromosomes from 4× Col-0 decreased compared with that from Col-0 Arabidopsis.

Supplementary Figure 4

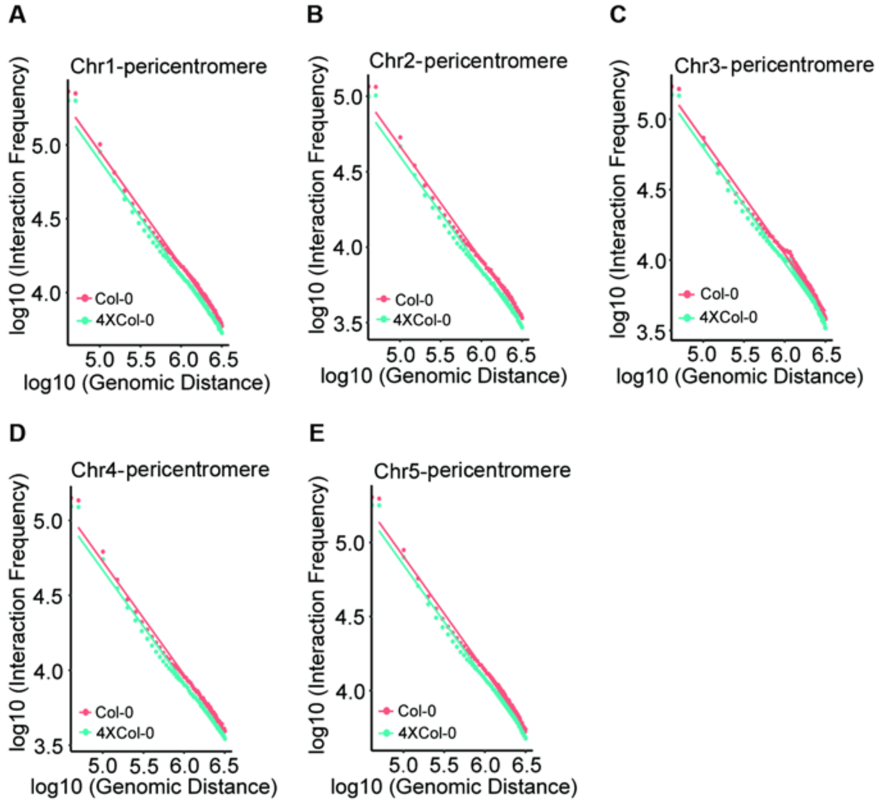

Supplementary Figure 4. Interaction decay exponents of pericentromeric regions of Col-0 and 4×Col-0. (**A-E**) Interaction decay exponents of pericentromeric regions of chr1, chr2, chr3, chr4 and chr5, respectively. The interaction frequency in pericentromeric regions from 4×Col-0 decreased compared with that from Col-0.

Supplementary Figure 5

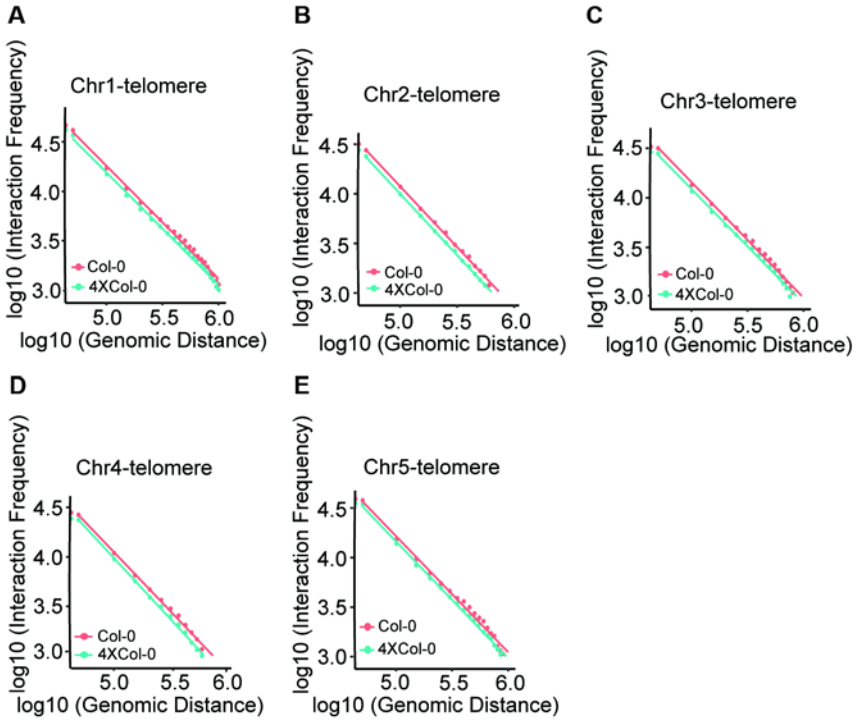

Supplementary Figure 5. Interaction decay exponents of telomeric regions of Col-0 and 4×Col-0. (**A-E**) Interaction decay exponents of telomeric regions in chr1, chr2, chr3, chr4 and chr5, respectively. The interaction frequency among telomeric regions in the 4×Col-0 decreased compared with that in Col-0.

Supplementary Figure 6

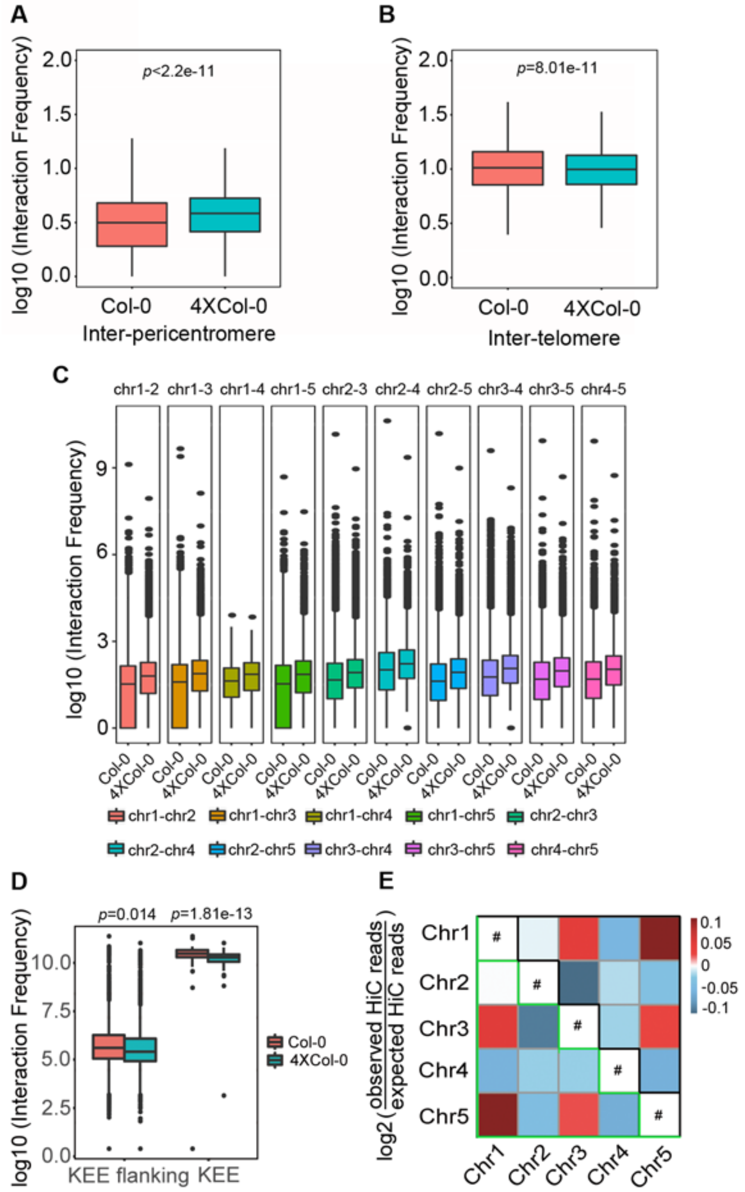

Supplementary Figure 6. Contact probability of pericentromeres, telomeres and chromosomal neighborhoods between Col-0 and 4×Col-0 Arabidopsis. (**A**) The interaction frequencies of inter-pericentromeres in Col-0 and 4×Col-0. (**B**) The interaction frequencies of inter-telomeres in Col-0 and 4×Col-0. (**C**) Boxplots showing inter-chromosome interaction frequencies among all chromosome pairs. (**D**) Interaction frequency of Knot Engaged Element (KEE) sites and flanking regions in Col-0 and 4×Col-0. (**E**) Features of chromosomal neighborhood were presented with log2 ratio of the observed to expected pair wise inter-chromosomal interactions between Col-0 and 4×Col-0. The upper black triangular matrix was 4×Col-0 and the lower green triangular matrix was Col-0 Arabidopsis. # means no values. The *p* values were calculated by Wilcoxon rank-sum test.

Supplementary Figure 7

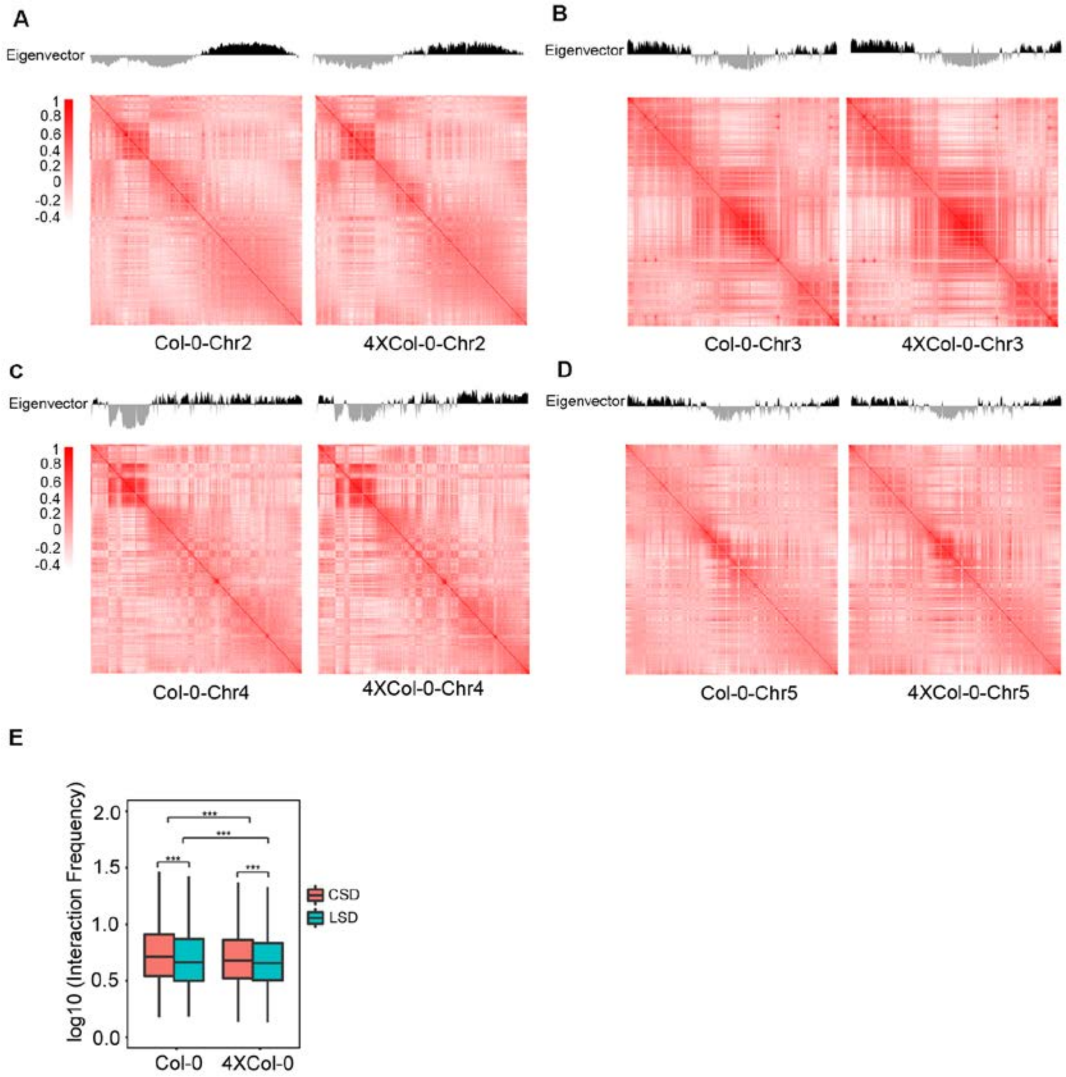

Supplementary Figure 7. Chromatin structure domains and interaction frequency in Col-0 and 4×Col-0 Arabidopsis. (**A-D**) Hi-C heatmaps of chromosome 2, 3, 4, and 5, respectively. The upper panel is Eigenvector representing LSD and CSD with positive and negative values respectively. (**E**) Boxplot showing a higher chromatin interaction frequency in CSD (brick red) and lower in LSD (blue). The *p* values were tested by Wilcoxon rank-sum test. ***represents *p* value<0.001.

Supplementary Figure 8

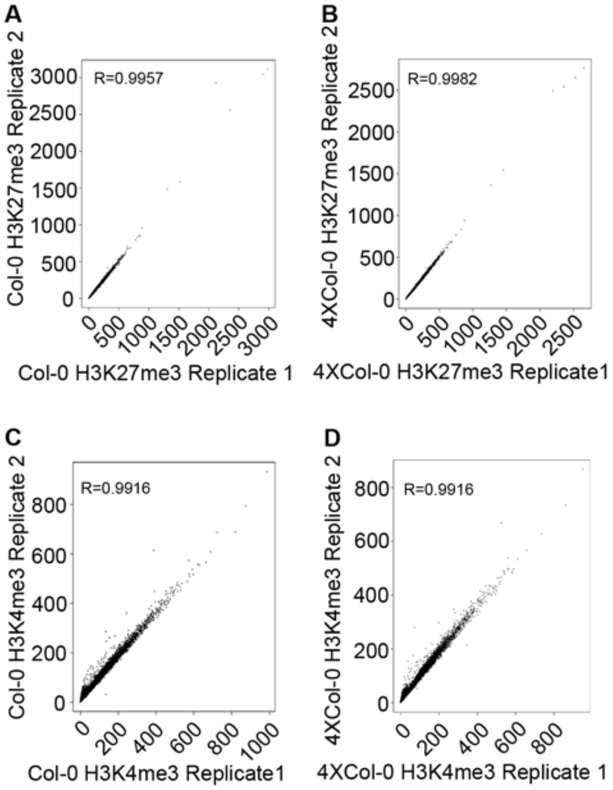

Supplementary Figure 8. Reproducibility analysis of H3K27me3 and H3K4me3 ChIP-seq replicates of Col-0 and 4×Col-0. (**A-B**) Scatter plots of two H3K27me3 ChIP-seq biological replicates using an anti-H3K27me3 antibody in Col-0 (**A**) and 4×Col-0 (**B**). (**C-D**) Scatter plots of two H3K4me3 ChIP-seq biological replicates using an anti-H3K4me3 antibody in Col-0 (**C**) and 4×Col-0 (**D**).

Supplementary Figure 9

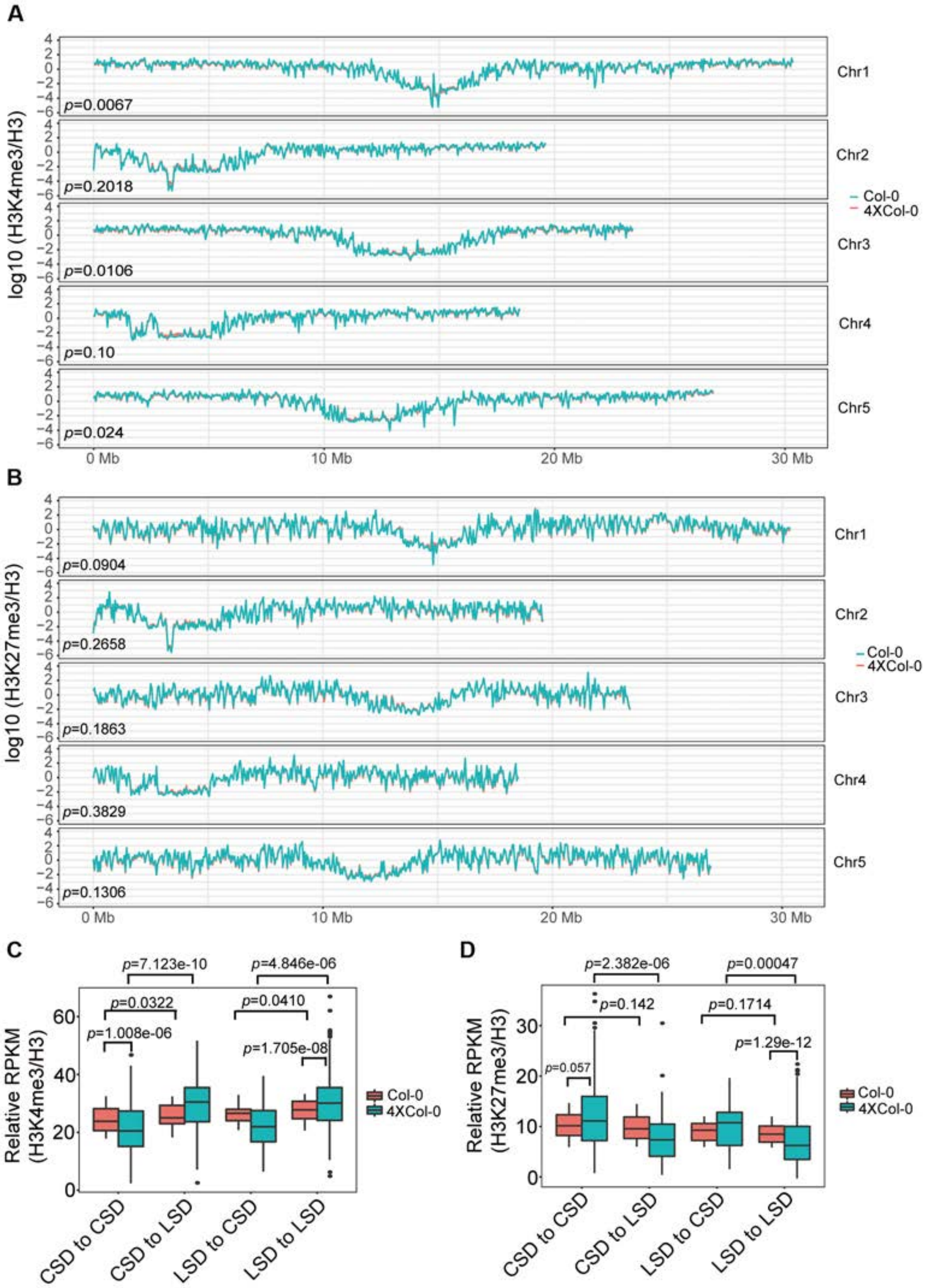

Supplementary Figure 9. Histone modifications in each chromosome and chromatin structure domains in Col-0 and 4×Col-0. (**A**) Distribution of the H3K4me3 marker along 5 chromosomes in Col-0 and 4×Col-0 Arabidopsis, plotted as the log of normalized H3K4me3 reads using H3 as the background. (**B**) Distribution of the H3K27me3 marker along 5 chromosomes in Col-0 and 4×Col-0 Arabidopsis, plotted as the log of normalized H3K27me3 reads using H3 as background. (**C**) Boxplot showing H3K4me3 changes at the converted or non-converted chromatin structure domains in autotetraploid compared with diploid Arabidopsis. (**D**) Boxplot showing H3K27me3 changes at the converted or non-converted structure domains in autotetraploid compared with diploid Arabidopsis.

Supplementary Figure 10

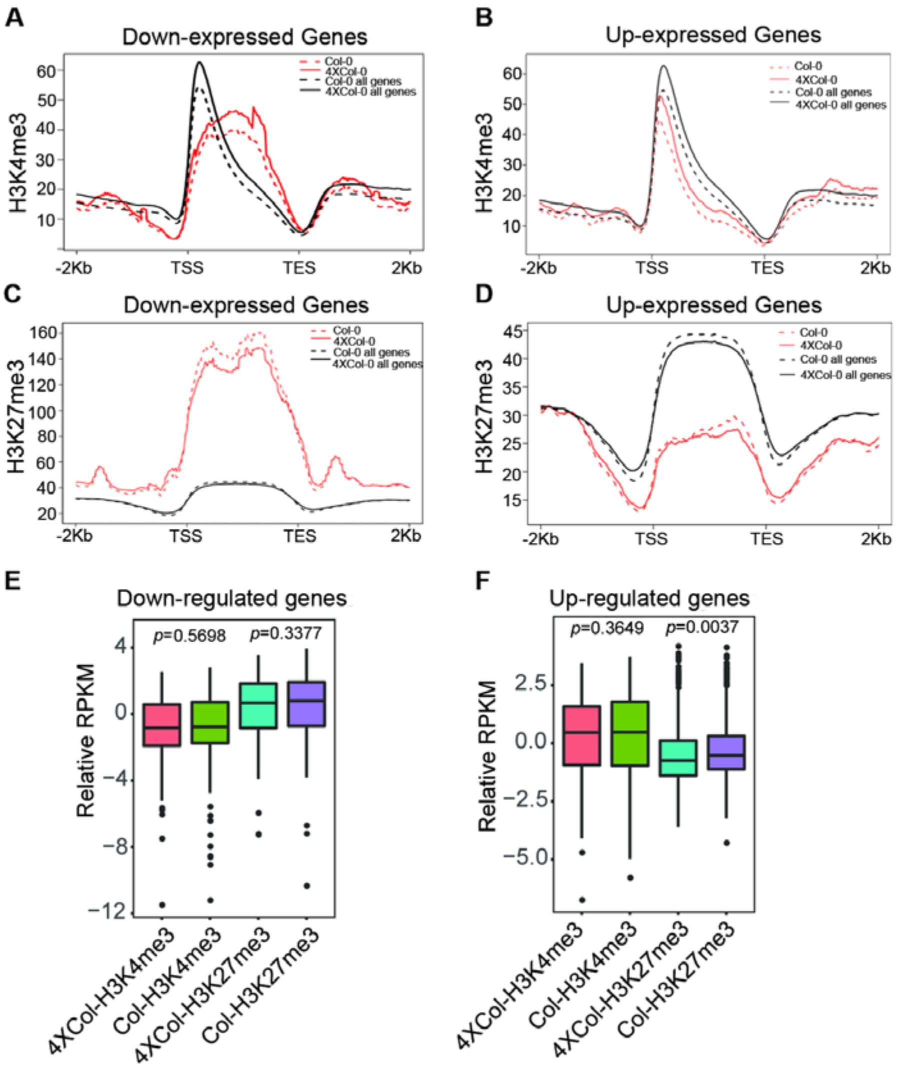

Supplementary Figure 10. Comparison the histone modification levels at the differentially expressed genes between Col-0 and 4×Col-0. (**A-B**) Comparison of H3K4me3 level in down-regulated (**A**) and up-regulated genes (**B**) between Col-0 and 4×Col-0. The black solid and dash curves indicate the average H3K4me3 modification in Col-0 and 4×Col-0. (**C-D**) Comparison of H3K27me3 level in down-regulated (**C**) and up-regulated genes (**D**) between Col-0 and 4×Col-0. The black solid and dash curves indicate the average H3K27me3 level in Col-0 and 4×Col-0. The down-regulated genes have a higher H3K27me3 level than all genes over gene bodies (TSS to TES) and up-regulated genes have a lower H3K27me3 level than all genes in both wild type and autotetraploid. (**E**) The relative amounts of H3K4me3 and H3K27me3 in down-regulated genes between Col-0 and 4×Col-0. These genes have similar levels of H3K4me3 and H3K27me3 in Col-0 and 4×Col-0. (**F**) The relative amounts of H3K4me3 and H3K27me3 in up-regulated genes between Col-0 and 4×Col-0. These genes show similar H3K4me3 level and slightly different H3K27me3 level in Col-0 and 4×Col-0. The p values were tested by Wilcoxon rank-sum test.

Supplementary Table 1. List of the differentially expressed genes between Col-0 and 4×Col-0 Arabidopsis.

Supplementary Table 2. GO terms of the differentially expressed genes in 4×Col-0 compared to Col-0.

Supplementary Table 3. The quality of Hi-C reads in Col-0 and 4×Col-0.

Supplementary Table 4. The Jaccard index between SD and histone modification.

Supplementary Table 5. The 539 common genes in differential interaction bins with differentially expressed genes between Col-0 and 4×Col-0 Arabidopsis.

Supplementary Table 6. The primers used in this work.
