# Supplementary material for: The effects of Arabidopsis genome duplication on the chromatin organization and transcriptional regulation
Source: Nucleic Acids Res. 2019 Jun 11;47(15):7857–69. doi: 10.1093/nar/gkz511 (PMC6736098; doi:10.1093/nar/gkz511)
Supplement: gkz511_Supplemental_Files [file gkz511_supplemental_files.zip › Supplementary Table 4 The Jaccard index between SDs and histone modification.docx]

The Jaccard Index between SD and histone modification

| Jaccard index | CSD to CSD | CSD to LSD | LSD to LSD | LSD to CSD |
| --- | --- | --- | --- | --- |
| H3K4me3 | 0.6702 | 0.6852 | 0.6179 | 0.6800 |
| H3K27me3 | 0.5290 | 0.6885 | 0.6841 | 0.6538 |
